# G-Protein-Coupled Receptor 91-Dependent Signalling Does Not Influence Vascular Inflammation and Atherosclerosis in Hyperlipidaemic Mice

**DOI:** 10.3390/cells12212580

**Published:** 2023-11-06

**Authors:** Silke Griepke, Mette Trauelsen, Michelle D. Nilsson, Jakob Hansen, Lasse B. Steffensen, Thue W. Schwartz, Daniel F. J. Ketelhuth

**Affiliations:** 1 Department of Cardiovascular and Renal Research, Institute of Molecular Medicine, University of Southern Denmark, 5000 Odense, Denmark; sgnielsen@health.sdu.dk (S.G.); duen@health.sdu.dk (M.D.N.); jakobhansen@health.sdu.dk (J.H.); lsteffensen@health.sdu.dk (L.B.S.); 2 Novo Nordisk Foundation Center for Basic Metabolic Research, University of Copenhagen, 2200 Copenhagen, Denmark; trauelsen@sund.ku.dk (M.T.); tws@sund.ku.dk (T.W.S.); 3 Center for Molecular Medicine, Department of Medicine, Karolinska Institutet, Bioclinicum, Solna, 171 64 Stockholm, Sweden

**Keywords:** atherosclerosis, inflammation, GPR91, SUCNR1, succinate, TCA, immunometabolism

## Abstract

The TCA cycle intermediate metabolite ‘succinate’ has been proposed as an inflammatory mediator, influencing autoimmunity and allergic reactions, through ligation to its sensing receptor SUCNR1/GPR91. Whether GPR91-mediated signalling influences the chronic inflammatory process of atherosclerosis has never been investigated. The examination of publicly available datasets revealed that the *SUCNR1* gene is expressed in human atherosclerotic plaques, especially in vascular smooth muscle cells. Using GPR91 knockout (*Gpr91−/−*) and wildtype (WT) littermates, made hyperlipidaemic with the overexpression of the gain-of-function mutated *Pcsk9* and Western diet feeding, we showed that the full ablation of GPR91 did not accelerate atherosclerosis—lesions in the aortic arch 2.18 ± 0.48% vs. 1.64 ± 0.31%, and in the aortic roots 10.06 ± 0.91% vs. 10.67 ± 1.53% for *Gpr91−/−* and WT mice, respectively. In line with this, no differences between groups were observed for macrophage and T-cell infiltration in the plaque, as well as the polarization towards M1- or M2-like macrophages in the aorta, spleen and liver of *Gpr91−/−* and WT control mice. In conclusion, our study indicates that the global ablation of GPR91 signalling does not influence vascular inflammation or atherogenesis.

## 1. Introduction

Atherosclerosis, the major cause of cardiovascular diseases (CVDs), including stroke and myocardial infarction, is a chronic inflammatory process affecting large- and medium-sized arteries [1]. This disease is initiated by the accumulation and modification of low-density lipoprotein (LDL) within the artery wall which triggers maladaptive innate and adaptive immune responses and drives the formation of atherosclerotic lesions [2]. As part of the atherosclerotic process, a fibrous cap that separates the artery lumen from the thrombogenic core of the plaque is usually formed. The rupture of this fibrous cap and superimposed thrombosis drive the most lethal consequences of CVDs [3].

Novel insights into the cellular processes driving inflammation have revealed that intrinsic cellular metabolism can reflect the activation, differentiation status and functional role of immune cells [4,5,6,7]. Proinflammatory cells such as M1-like macrophages rely strongly on aerobic glycolysis and lactate formation and have their tricarboxylic acid (TCA) cycle disrupted at different steps, resulting in the accumulation of certain metabolites, especially succinate [8]. Differently, anti-inflammatory cells such as M2-like macrophages display a distinct metabolic profile, characterized by a functional and intact TCA cycle, increased oxidative phosphorylation (OXPHOS) and efficient ATP production [9].

While for many years, metabolites have been considered as merely building blocks of energy production, recent research has revealed additional metabolite-mediated functions, including major roles in post-translational modification, epigenetic alterations, and receptor-dependent signalling functions [10,11,12]. A well-characterized example of a metabolite affecting immune cell function is the intracellular accumulation of succinate on proinflammatory/activated macrophages, which has been shown to prevent hydroxylation and stabilize hypoxia-inducible factor-1-alpha (HIF1α)-mediated signalling [8]. The stabilization and translocation of HIF1α to the nucleus can influence both metabolic and inflammatory functions, including the overexpression of glycolytic and interleukin-1 beta (IL-1β) genes in macrophages [8].

The conditions that drive succinate accumulation, for example, hypoxia or inflammation, also increase its extracellular levels, allowing it to exert autocrine and paracrine functions on its sensing receptor, the G-protein-coupled receptor 91 (GPR91 or SUCNR1). Within the immune system, GPR91 is expressed in macrophages, immature dendritic cells and mast cells [13,14,15], and has also been implicated in the modulation of IL-1 secretion [14].

Several immunomodulatory strategies have been proven to ameliorate CVD in animal models and humans [16,17,18,19]. Thus, we and others have shown that the modulation of the metabolism of immune cells can substantially influence vascular inflammation and experimental atherosclerosis [20,21,22,23,24,25,26,27]. Several GPCRs have been identified to elicit signal transduction when triggered by extracellular metabolites, including intermediate metabolites of the TCA cycle, triglycerides, bile acids and amino acids [28]. Many of these GPCRs have been linked with immunoregulation, including the fatty acid receptors GPR120, GPR40, GPR84, GPR41 and GPR43; the receptors for 3-hydroxy-butyrate/niacin GPR109A; the receptor for 3-hydroxy octanoate GPR109B; the receptor for lactate GPR81; the receptor for kynurenic acid GPR35; and the succinate receptor GPR91 [29]. Despite this knowledge, only a few of these receptors have been investigated in the context of atherosclerosis.

Triggering GPR91 signalling has been implicated in several inflammatory diseases such as inflammatory bowel disease, rheumatoid arthritis and asthma [30,31]. Interestingly, elevated plasma succinate levels have been linked to increased CVD risk in young individuals [32], and repeated injections of succinate have been shown to accelerate atherosclerosis in *Apoe−/−* mice [33]. Despite this previous knowledge, whether GPR91-mediated signalling plays a role in the pathophysiology of atherosclerosis remains unknown. In the present study, the induction of hyperlipidaemia and atherosclerosis in *Gpr91−/−* and WT littermate mice, with the injection of recombinant adeno-associated virus vector (AAV) encoding gain-of-function mutant *Pcsk9*, showed that the overall ablation of GPR91-dependent signalling does not influence vascular inflammation and atherogenesis.

## 2. Materials and Methods

### 2.1. GPR91 Expression in the Human Vasculature—Analyses of Public Datasets

*GPR91* (*SUCNR1*) transcript levels in healthy post mortem tissue samples (heart left ventricle (*n* = 432), heart atrial appendage (*n* = 429), tibial artery (*n* = 663), coronary artery (*n* = 240), aorta (*n* = 432), liver (*n* = 226) and spleen (*n* = 241)) were obtained from the Genotype-Tissue Expression (GTEx) portal V8 [34]. Cell-specific GPR91 (*SUCNR1*) expression was obtained using publicly available single-cell RNA sequencing (scRNA-seq) datasets from human carotid (GSE155512 [35], GSE159677 [36]) and coronary atherosclerotic plaques (GSE131778 [37]). All datasets underwent QC analysis and exploration, which were performed using the R package Seurat 4.3.0 [38]. Parameters used for filtering and clustering are shown in the Supplementary Materials (Supplementary Table S2). Cell clusters were identified using an optimized published pipeline [39]; cell clusters were annotated using integration and reference-based approaches using the R package SingleR and the reference datasets “BlueprintEncodeData” and “HumanPrimaryCellAtlasData” from the celldex R package. Integration-based cluster annotation was performed using the Seurat package and the vasculature scRNAseq dataset from Tabula Sapiens [40]. Cluster annotations were validated by manual differential expression analysis. The expression of *SUCNR1/GPR91* was shown across cell clusters for each dataset.

### 2.2. Animal Model and Experimental Design

*Gpr91−/−* mice, generated by the replacement of part of exon 2 (5′-GGCTACCTCTTCTGCAT-3′) with a lacZ-neomycin cassette [13] and by 10 generations backcrossed with C57BL6/J [11] (kindly provided by Amanda Littlewood-Evans, Novartis Institutes for BioMedical Research, Basel, Switzerland), were established at the Department of Experimental Medicine, University of Copenhagen, Denmark. Breeding colonies were maintained as heterozygous (*Gpr91+/−*) for breeding to obtain littermate wildtype (WT) and *Gpr91−/−* mice. At 10 weeks of age, male WT and *Gpr91−/−* mice were made hyperlipidaemic with an intravenous injection of 1011 viral genomes of rAAV8-D377Y-mPcsk9 and concomitant feeding with a Western-type diet (WTD, 21% fat, 0.21% cholesterol; D12079B, Research Diets Inc., New Brunswick, NJ, USA) for 14 weeks, as previously described [41]. On every second week, mice were weighed, and blood was harvested from the facial vein to monitor plasma cholesterol levels. At the endpoint, mice were euthanized through exposure to saturated isoflurane vapours. All animal experiments were conducted according to the guidelines of Directive 2010/63/EU from the European Parliament on the protection of animals used for scientific purposes and approved by the Danish Animal Experiments Inspectorate (2018-15-0201-01424).

### 2.3. Tissue Collection and Analyses of Atherosclerotic Lesions

After sacrifice, blood from WT and *Gpr91−/−* was collected by cardiac puncture and vascular perfusion was performed with phosphate-buffered saline (PBS). After perfusion, the heart and aortic arch were dissected and saved for later lesion and immunohistochemistry analyses. En face lipid accumulation was measured in 4% paraformaldehyde (PFA)-fixed aortic arches using Sudan IV (Sigma-Aldrich, St. Louis, MO, USA) staining, as previously described [25]. The lesion area was calculated as the sum of the total lesion area in the aortic arch (excluding the branching vessels). Aortic root lesions were evaluated in cryopreserved hearts that were serially sectioned from the proximal part of the aortic root on a cryostat. Lesion size was measured from Oil Red O (ORO)- and haematoxylin-stained PFA-fixed 10 μm sections, collected every 100 μm over an 800 μm segment of the aortic root starting at the beginning of the cusps. The lesion size of the ORO-stained aortic root sections was quantified as previously described [42]. Samples that were compromised during processing were excluded from the study. The evaluation of lesions was performed by trained personnel who were blinded to the mouse genotypes.

The cellular compositions of the aortic root lesions were evaluated by staining 10 μm thick acetone-fixed sections serially taken from cryopreserved hearts. Primary antibodies directed against the macrophage marker CD68 (Bio-Rad Laboratories, Hercules, CA, USA), T-cell marker CD3 (Abcam, Cambridge, UK), the marker of activation VCAM-1 (BD Biosciences, Franklin Lakes, NJ, USA), MHC class II marker I-Ab (BD Biosciences, Franklin Lakes, NJ, USA) and vascular smooth muscle cell (VSMC) marker alpha-smooth muscle actin (αSMA) (Abcam, Cambridge, UK) were used for visualization. Primary antibodies were detected using biotinylated anti-rat IgG (Dako, Glostrup, Denmark) or anti-rabbit IgG (Vector Laboratories, Burlingame, CA, USA). Staining reactions were developed using the VEC-TASTAIN ABC kit (Vector Laboratories, Burlingame, CA, USA) and diaminobenzidine (DAB) (Dako, Glostrup, Denmark). αSMA was detected using standard horseradish peroxidase (HRP)-conjugated anti-rabbit antibody (Dako, Glostrup, Denmark) followed by DAB (Dako, Glostrup, Denmark). Nuclei were counterstained with Mayer’s haematoxylin (Sigma-Aldrich, St. Louis, MO, USA). Samples that were compromised during processing were excluded from the study. The evaluation of lesions was performed by trained personnel who were blinded to the mouse genotypes using ImageJ software (National Institutes of Health, Bethesda, MD, USA).

### 2.4. Plasma Lipid Measurements

Plasma cholesterol and triglyceride levels were measured using enzymatic colourimetric kits (CHOL2 and TRIGL, Roche, Basel, Switzerland), according to the manufacturer’s instructions.

### 2.5. Succinate Analysis

Succinate levels in plasma samples were evaluated using a classic colourimetric assay (Sigma-Aldrich, St. Louis, MO, USA), following the manufacturer’s instructions.

### 2.6. RNA Isolation and Analysis

RNA from spleens and liver was isolated in TRIzol solution (Thermo Fisher Scientific, Waltham, MA, USA) by homogenization using a tissue Lyser II and a 5 mm stainless steel bead (Qiagen, Hilden, Germany), followed by RNA phase separation using chloroform and RNA precipitation using isopropanol. RNA from the aortas was isolated using a total RNA purification Kit (Norgen Biotek Corp., Thorold, ON, Canada) following the manufacturer’s instructions. RNA quality was approved on a Nanophotometer (Implen GmbH, München, Germany) followed by the reverse transcription of 1μg of total RNA using an iScriptTM cDNA synthesis kit (Bio-Rad Laboratories, Hercules, CA, USA). Quantitative gene expression analyses were performed by quantitative real-time PCR using a TaqMan assay on demand for *Gpr91* (Thermo Fisher Scientific, Waltham, MA, USA); and specific primers for *Cd68*, *Tnf*, *Ccl2*, *Cxcl10*, *Ccr1*, *Nos2*, *Cd206*, *Arg1*, *Chil3* and *Fizz1* using a SYBR-green detection system (Bio-Rad Laboratories, Hercules, CA, USA). TATA-binding protein (*Tbp*) and Transcription factor II B (*TfIIb*) mRNA were used as housekeeping genes. A full list of primers used in the study is provided in Supplementary Table S1. Data were analysed based on the relative expression method with the formula 2^−ΔΔct^, where ΔΔct = Δct (sample) − Δct (calibrator = mean ct values of all samples within the WT group), and Δct is the mean ct-value of the housekeeping gene subtracted from the ct-value of the target gene.

### 2.7. Analysis of Biomarkers of Liver Damage

The hepatocellular enzyme aspartate aminotransaminase (AST) that is released into the blood upon hepatic cell damage was quantified using a colourimetric assay kit (Cayman Chemical, Ann Arbor, MI, USA), following the manufacturer’s instructions. Transcript levels of ATP Binding Cassette Subfamily C Member 6 (*Abcc6*) and G3BP Stress Granule Assembly Factor 1 (*G3bp1*), which have been proposed as biomarkers of liver injury [43], were quantified by qPCR in liver samples, as previously described.

### 2.8. Statistical Analysis

The results are presented as the mean ± standard error of the mean (SEM). The Mann–Whitney U-test was used for two group comparisons, and a two-way ANOVA with a Bonferroni post hoc test was applied to test differences between groups over time. In all analyses, differences were considered significant at *p*-values < 0.05 (two-tailed). All statistical analyses of in vivo and in vitro experiments were performed using GraphPad Prism version 9.3.1 (GraphPad Software Inc., San Diego, CA, USA).

## 3. Results

### 3.1. SUCNR1/GPR91 Is Expressed in Vascular and Immune Cells from Human Atherosclerotic Plaques

**Figure 1:**
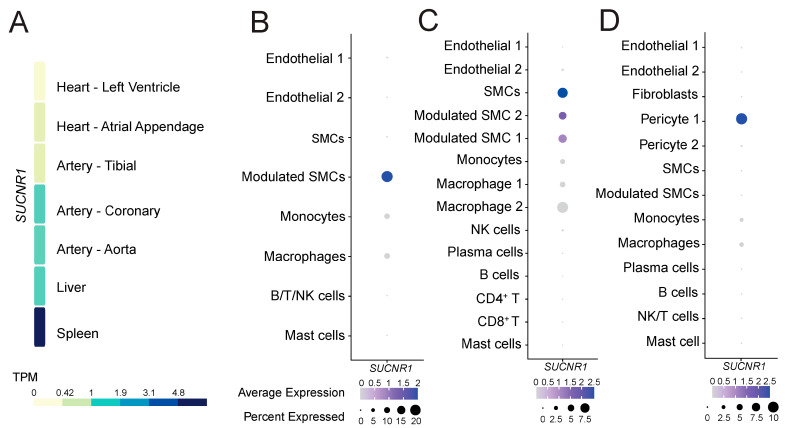
The expression pattern of *SUCNR1/GPR91* in human tissues and atherosclerosis. (**A**) Data from the GTEx resource portal expressed as transcripts per million (TPM). (**B**–**D**) Dot plots depict mRNA levels of SUCNR1/GPR91 in common vascular and immune cell populations. Dot size depicts the percentage fraction of cells expressing a gene, and dot colour intensity depicts the degree of expression of each gene. (**B**) Data from carotid plaques, *n* = 18; (**C**) data from carotid plaques, *n* = 3; (**D**) data from coronary plaques, *n* = 4 [35,36,37]. SMC; smooth muscle cell, NK; natural killer cell; T, T lymphocytes; B, B lymphocytes.

To gain insight into the role of GPR91 in CVDs, we explored whether the receptor would be expressed in healthy cardiovascular and atherosclerotic disease tissues. Analysis of the GTEx data of healthy tissues revealed that *SUCNR1*/*GPR91* is more abundantly expressed on the transcript level in the human coronary artery and the aorta, and to a lesser extent in the tibial artery and heart muscle (Figure 1A). Notably, *SUCNR1*/*GPR91* expression in the cardiovascular system seemed 4- to 5-fold lower than that found in human spleen (Figure 1A)—the latter signal is likely to be driven by *SUCNR1*/*GPR91* expression in macrophages within the organ (Supplementary Figure S1).

Next, we enquired whether *SUCNR1*/*GPR91* is also expressed in human atherosclerotic tissue, and by which cells. Analyses of three public datasets of scRNA-Seq [35,36,37] suggest *SUCNR1*/*GPR91* is expressed especially in VSMC populations, pericytes and also by macrophages (Figure 1B–D). Notably, the *SUCNR1*/*GPR91* signal is higher in the vascular cells compared to the immune cells (Figure 1B–D).

### 3.2. Hyperlipidaemic WT and Gpr91−/− Mice Present Similar Plasma Lipid Levels and Atherosclerosis Burden

**Figure 2:**
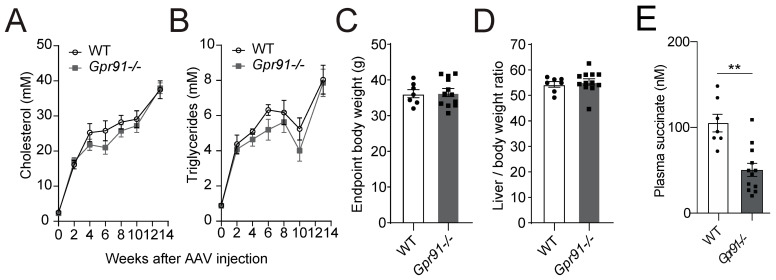
Plasma lipid levels and atherosclerotic burden in hyperlipidaemic WT and *Gpr91−/−* mice. Mice were injected with rAAV8-D377Y-mPcsk9 and fed a WTD for 14 weeks. (**A**) Cholesterol levels and (**B**) triglyceride levels in the plasma of WT and *Gpr91−/−* mice (*n* = 7–12). (**C**) Total body weight, (**D**) liver-to-body weight ratio at the end of the experiment and (**E**) plasma succinate levels in the plasma of WT and *Gpr91−/−* mice (*n* = 7–12). Results are shown as mean ± SEM; graphs show pooled data from two independent experiments. (**A**,**B**) Two-way ANOVA with Bonferroni post hoc test; (**C**,**D**,**E**) Mann–Whitney U-test. **: *p* < 0.01.

Analyses of serially collected blood from *Gpr91−/−* mice and WT littermate controls, which were injected with rAAV8-D377Y-mPcsk9 and fed a WTD for 14 weeks, confirmed the expected development of hypercholesterolaemia–in both groups, an approximately 8-fold increase in plasma cholesterol and a 4-fold increase in triglycerides was observed after 2 weeks of injection and diet, and 19-fold increased cholesterol and 8-fold increased triglycerides levels were observed at the endpoint. Notably, the latter findings replicated the seminal work describing the AAV-induced hyperlipidaemia model well [44]. Hence, mice carrying the genetic deficiency on *Gpr91* showed very similar kinetics of increases in plasma lipids, and no difference against WT controls was observed (Figure 2A,B), and no significant differences in the total body weight and liver-to-body weight ratio were observed between the groups (Figure 2C,D). Surprisingly, succinate levels were found to be decreased by approximately half in plasma from *Gpr91−/−* mice versus the control (Figure 2E).

**Figure 3:**
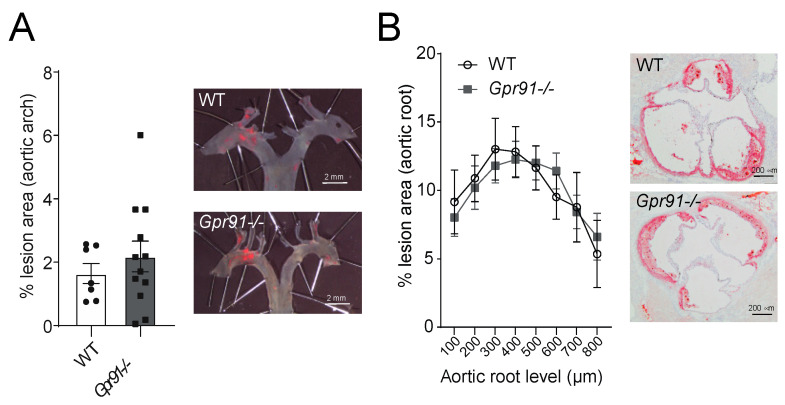
Effects of *Gpr91* genetic ablation on atherosclerosis development. Mice were injected with rAAV8-D377Y-mPcsk9 and fed a WTD for 14 weeks. (**A**) Quantification of en face Sudan IV-stained aortic arches from WT and *Gpr91−/−* mice (*n* = 7–12); right panes show representative micrographs of the atherosclerotic burden. (**B**) Quantification of atherosclerotic ORO-stained proximal aortic root lesions shown as % lesion areas of total aortic root area (*n* = 7–12); right panes show representative micrographs of the atherosclerotic burden. Results are shown as mean ± SEM; graphs show pooled data from two independent experiments. No significant differences were observed. (**A**) Mann–Whitney U-test; (**B**) two-way ANOVA with Bonferroni post hoc test.

Next, we used dissected aortic arches and sections from the aortic root to evaluate the impact of GPR91 signalling in vascular inflammation and atherosclerotic burden. Hypercholesterolaemia induced by the injection of rAAV8-D377Y-mPcsk9 and WTD feeding allowed both groups to develop advanced atherosclerotic lesions (Figure 3). However, no significant difference in the burden of atherosclerosis was observed between groups, including the percentage of lesions in the aortic arches (Figure 3A) and ORO-stained lesions in the aortic root (Figure 3B).

### 3.3. Genetic Ablation of GPR91-Mediated Signalling Does Not Influence Vascular Inflammation

**Figure 4:**
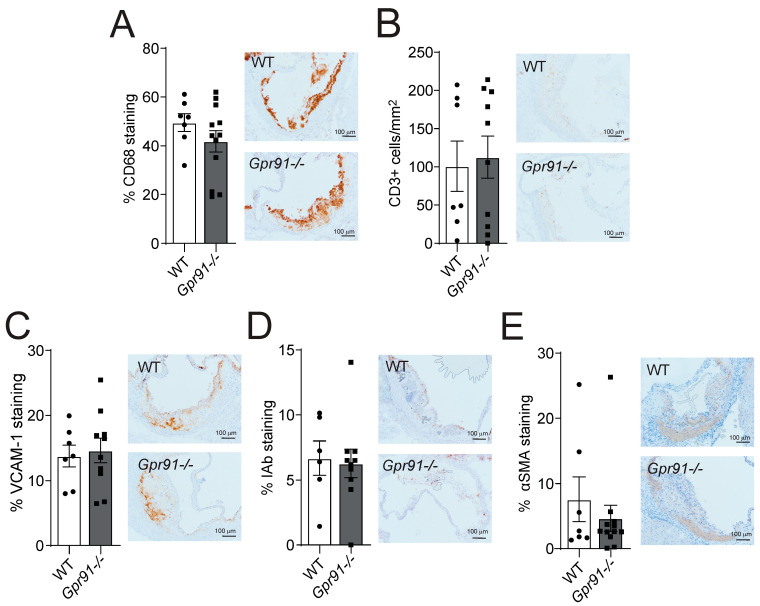
Effects of *Gpr91* genetic ablation on plaque inflammatory cell composition. Mice were injected with rAAV8-D377Y-mPcsk9 and fed a WTD for 14 weeks. (**A**) Percentage of CD68+ macrophages, (**B**) the number of CD3+ T cells /mm^2^, (**C**) the percentage of VCAM-1+ stained cells, (**D**) the percentage of IAb+ cells and (**E**) the percentage of α-smooth muscle cell actin (αSMA) staining in aortic root lesions from WT and *Gpr91−/−* mice (*n* = 6–12). The right panels show representative micrographs of stained sections from each group. Results are shown as mean ± SEM; graphs show pooled data from two independent experiments. No significant differences were observed; Mann–Whitney U-test.

Immunohistochemical staining of main inflammatory markers in the aortic root sections revealed that the global ablation of GPR91-mediated signalling did not impact the percentage of infiltrating CD68+ macrophages, the number of infiltrating CD3+ T-cells, the percentage of adhesion molecule VCAM-1 expression and MHC-II IAb+ cells, as well as the percentage of αSMA+ VSMCs compared to WT mice (Figure 4A–E).

**Figure 5:**
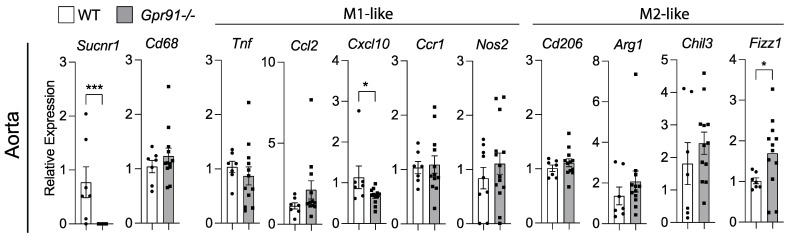
Expression of macrophage polarization markers in the aorta of WT and *Gpr91−/−* mice. Mice were injected with rAAV8-D377Y-mPcsk9 and fed a WTD for 14 weeks. Relative mRNA expression of *Sucnr1*/*Gpr91*, *Cd68*, *Tnf*, *Ccl2*, *Cxcl10*, *Ccr1*, *Nos2*, *Cd206*, *Arg1*, *Chil3* and *Fizz1* in the aorta from WT and *Gpr91−/−* mice (*n* = 7–12). Results are shown as mean ± SEM; graphs show pooled data from two independent experiments. * *p* < 0.05 and *** *p* < 0.001. Mann–Whitney U-test.

Because our bioinformatic analysis of human plaques suggested that SUCNR1/GPR91 can be expressed by macrophages, we investigated transcript markers of macrophage polarization in the aorta of our mice. In line with the previous data, the analysis of aortic transcripts for *Cd68* showed no difference between groups (Figure 5). Although the M1-like marker *Cxcl10* was found reduced and the M2-like marker *Fizz1* increased in *Gpr91−/−* mice (Figure 5), no clear polarization of macrophages seemed to occur upon the ablation of GPR91-mediated signalling, and no other marker tested showed differences between groups.

### 3.4. Macrophage Polarization in the Spleen and Liver Is Not Clearly Affected by GPR91 Ablation

**Figure 6:**
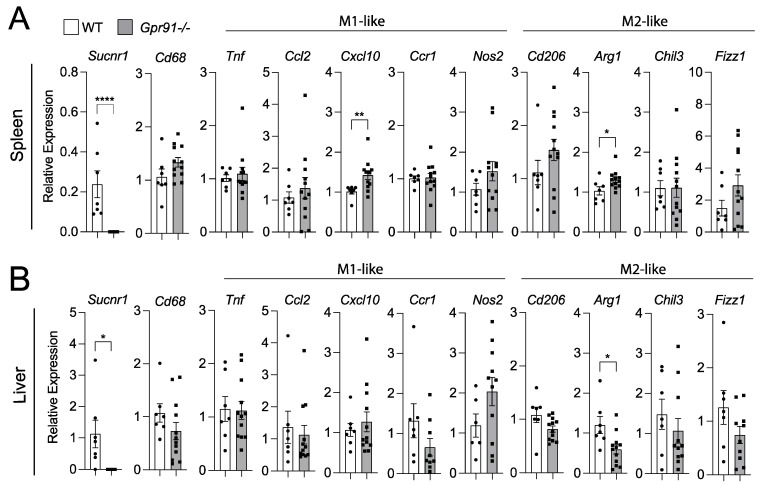
Expression of macrophage polarization markers in the spleen and liver of WT and *Gpr91−/−* mice. Mice were injected with rAAV8-D377Y-mPcsk9 and fed a WTD for 14 weeks. Relative mRNA expression of *Gpr91*, *Cd68*, *Ccl2*, *Cxcl10*, *Ccr1*, *Cd206*, *Arg1*, *Chil3* and *Fizz1* in (**A**) the spleen and (**B**) the liver from WT and *Gpr91−/−* mice (*n* = 7–12). Results are shown as mean ± SEM; graphs show pooled data from two independent experiments. * *p* < 0.5, ** *p* < 0.01, **** *p* < 0.0001. Mann–Whitney U-test.

Considering our bioinformatic analysis indicated increased *SUCNR1* expression in the spleen compared to vascular tissues and liver, we decided to also investigate transcript markers of macrophage polarization in these organs. Although we observed a trend towards the increased splenic expression of *Cd68* in *Gpr91−/−* mice versus controls, no clear pattern towards M1- or M2-like polarization was seen between groups (Figure 6A). 

Notably, the M1-like marker *Cxcl10* and the M2-like marker *Arg1* were found to be increased in the spleen of *Gpr91−/−* mice (Figure 6A). Similar analyses in the liver revealed the M2-maker *Arg1* to be downregulated in *Gpr91−/−* mice, while the expression of all the other markers showed no difference between groups (Figure 6B). Of note, *Sucnr1* expression was confirmed to be neglectable in the aorta, spleen and liver from *Gpr91−/−* mice and detected in WT mice (Figure 5 and Figure 6). Moreover, no difference in plasma AST levels and the transcript markers associated with liver damage, *Abcc6* and *G3bp1* [43], were seen between groups (Supplementary Figure S2).

## 4. Discussion

Recent research indicates that intrinsic alterations in cellular metabolism coincide with the exacerbation of vascular inflammation and increased risk of developing cerebrovascular events [45]. In this context, it has been shown that plaques at a high risk of rupture present a skewed metabolic signature with increased glycolysis, elevated amino acid utilization and decreased FAO—a similar profile to that found in immune pro-inflammatory and cancer cells [45,46]. Despite previous evidence indicating that increased succinate signalling in the vascular wall could positively influence atherogenesis [27], in this study, we showed that the ablation of signalling through the succinate receptor GPR91 does not influence vascular inflammation and atherosclerotic plaque burden in hyperlipidaemic mice.

Atherogenesis involves complex crosstalk between different organs and cells throughout the body. Hence, vascular inflammation, the development of atherosclerotic plaques, and the potential progression into an unstable plaque phenotype are the result of the intricate interplay between vascular and immune cells in the artery and paracrine and endocrine “signals” from various cells and organs, including those involved in metabolic regulation such as the gut, kidney, liver and adipose tissue [19]—all these organs have been shown to express SUCNR1/GPR91 [47,48,49].

As mentioned earlier, succinate levels have been associated with an increased risk of CVD [32]. In this study, analyses of public transcriptome datasets showed that *SUCNR1/GPR91* is present in the vasculature of humans, being expressed particularly by VSMCs/pericytes and to a lesser extent by macrophages within plaques, thus suggesting that in a paracrine and/or endocrine manner, succinate-mediated GPR91 signalling could influence cellular functions and disease. The fact that the succinate/GPR91 axis has been linked to pathological cardiomyocyte hypertrophy through the regulation of intracellular Ca2+ release and re-uptake by cardiomyocytes [50], as well as the control of blood pressure, via the regulation of renin release from kidneys and the secretion of nitric oxide and prostaglandin-E2 from endothelial cells [51], suggests that several parallel mechanisms that influence the course of atherogenesis could be influenced by this signalling pathway. Given that SUCNR1/GPR91 expression could be more abundant and may play a greater role in organs other than the vasculature, e.g., the intestine, liver and adipose tissue [28,52,53,54], the possibility that succinate-mediated GPR91 signalling may influence CVD indirectly via the regulation of immune and metabolic responses in these organs should not be disregarded.

Lukasova et al., 2011 showed that the lipid-lowering drug niacin has lipid-independent inhibitory effects on atherosclerosis mediated by GPR109A on macrophages [55]. Pols et al., 2011 showed that the activation of bile acid receptor TGR5 attenuates atherosclerosis, an effect that is lost in *Ldlr−/−Tgr5−/−* mice [56]; notably, the protective effects of TGR5 ligation on atherosclerosis was shown to be enhanced by the combined ligation of FXR in hyperglycaemic models [57]. Interestingly, while the former studies suggested that the targeting of metabolite receptors could be used to regulate vascular inflammation and prevent atherosclerosis, the ablation of GPR109A and TGR5 signalling alone, in the absence of concomitant treatment with an excess of specific agonists, did not result in the acceleration of disease [55,56]. Speculatively, these data suggest that the influence of metabolite-sensing GPCRs in atherogenesis may also depend on the abundance or excess of their specific metabolites, as well as the degree of their metabolism. The fact that we know injections of succinate accelerate experimental atherosclerosis [33], as well as our discovery of significantly lower levels of succinate in the plasma of hyperlipidemic *Gpr91−/−* mice implies that lesions could be reduced later on under similar experimental conditions. It will be interesting to explore in the future whether the potentially deleterious effects of an excess of succinate (e.g., with injections) can be mitigated by the ablation of GPR91, which could also shed light on the various mechanisms, both receptor dependent and receptor independent, activated in the artery wall. Thus, future research should also attempt to clarify how succinate secretion may be regulated by GPR91-dependent signalling.

It has been shown that the disruption of GPR120 or GPR35 signalling in bone marrow-derived cells does not affect vascular inflammation and atherosclerosis in *Ldlr−/−* mice [58,59]. Hence, the fact that no substantial changes were observed in the arteries of our hyperlipidaemic *Gpr91−/−* mice compared to controls, in spite of the concomitant reduction in plasma succinate levels, raises a few additional thoughts about the general role of metabolite-sensing GPCRs in the context of cardiometabolic diseases. The most important of them could be that GPR91 may play both pro- and anti-atherogenic roles depending on the cell expression specificity.

Previous studies showed that the inflammatory response of macrophages and dendritic cells from *Gpr91−/−* mice is substantially impaired [13,14], and so, succinate-signalling through GPR91 on immune cells has been implicated with fibrotic responses in the liver [60] and with the exacerbation of joint inflammation in the context of collagen-induced arthritis in mice [14]. However, in the adipose tissue, the same signalling pathway has been proposed to promote thermogenesis, improve tolerance to glucose and protect mice from diet-induced obesity [61]. Despite the limitations of our multi-organ analysis of inflammation, which primarily involved the assessment of non-exclusive cellular transcript markers of macrophage polarisation, and considering the multifactorial nature of the atherosclerotic disease, we speculate that competing systemic and local vascular effects could be a reason for the nearly null results observed in the vascular wall.

It has been suggested that GPR91 signalling has a “biphasic” role in adipose tissue, promoting obesity and insulin resistance in the early stages and having protective effects in the late stages of the disease [62]. Our experimental design evaluated the atherosclerotic burden with fourteen weeks of WTD feeding, a relatively early time point for this hyperlipidaemic model. In this context, another potential explanation for no major phenotype in our experiments with *Gpr91−/−* mice could be related to the stage of the disease, and whether GPR91 may affect the late stages of atherosclerosis warrants future investigation. Notably, PCSK9, which was overexpressed in our mice following an AAV injection, has been linked to both LDLR-dependent and -independent mechanisms that promote pro-inflammatory macrophage responses [63,64,65,66]. Although both groups in our study seemed to not exhibit exacerbated atherogenesis when compared to previous studies using rAAV-*mPcsk9* or *Ldlr−/−* mice [44], whether PCSK9 influences the succinate/GPR91 axis warrants further investigation.

## 5. Conclusions

In conclusion, we showed that *SUCNR1/GPR91* is expressed in healthy vasculature as well as atherosclerotic plaques in humans. Using an animal model of disease, we demonstrated that the ablation of GPR91 signalling does not significantly impact the atherosclerotic burden. Taking into account the current knowledge that GPR91 may play different cellular-specific roles in the spectrum of cardiometabolic diseases, future studies targeting this receptor, through ablation or overexpression, specifically in metabolic, vascular or immune cells, as well as the evaluation of disease at different stages, will be needed to provide definitive proof of the involvement of GPR91 signalling in the pathophysiological process of atherosclerosis. 

## Data Availability

Available on request from the corresponding author.

## Supplementary Materials

The following supporting information can be downloaded at: https://www.mdpi.com/article/10.3390/cells12212580/s1, Table S1: List of primers used in the study; Table S2: Filtering and parameters used for analyses of scRNAseq datasets; Figure S1: SUCNR1(GPR91) is expressed mainly in macrophages in the human spleen; Figure S2: Liver toxicity status.

## References

[1] Herrington W., Lacey B., Sherliker P., Armitage J., Lewington S. (2016). Epidemiology of Atherosclerosis and the Potential to Reduce the Global Burden of Atherothrombotic Disease. Circ. Res..

[2] Tabas I., Williams K.J., Boren J. (2007). Subendothelial lipoprotein retention as the initiating process in atherosclerosis: Update and therapeutic implications. Circulation.

[3] Bentzon J.F., Otsuka F., Virmani R., Falk E. (2014). Mechanisms of plaque formation and rupture. Circ. Res..

[4] Jha A.K., Huang S.C., Sergushichev A., Lampropoulou V., Ivanova Y., Loginicheva E., Chmielewski K., Stewart K.M., Ashall J., Everts B. (2015). Network integration of parallel metabolic and transcriptional data reveals metabolic modules that regulate macrophage polarization. Immunity.

[5] Gerriets V.A., Kishton R.J., Nichols A.G., Macintyre A.N., Inoue M., Ilkayeva O., Winter P.S., Liu X., Priyadharshini B., Slawinska M.E. (2015). Metabolic programming and PDHK1 control CD4+ T cell subsets and inflammation. J. Clin. Investig..

[6] Soriano-Baguet L., Grusdat M., Kurniawan H., Benzarti M., Binsfeld C., Ewen A., Longworth J., Bonetti L., Guerra L., Franchina D.G. (2023). Pyruvate dehydrogenase fuels a critical citrate pool that is essential for Th17 cell effector functions. Cell Rep..

[7] Forteza M.J., Ketelhuth D.F.J. (2022). Metabolism in atherosclerotic plaques: Immunoregulatory mechanisms in the arterial wall. Clin. Sci..

[8] Tannahill G.M., Curtis A.M., Adamik J., Palsson-McDermott E.M., McGettrick A.F., Goel G., Frezza C., Bernard N.J., Kelly B., Foley N.H. (2013). Succinate is an inflammatory signal that induces IL-1beta through HIF-1alpha. Nature.

[9] Palsson-McDermott E.M., O’Neill L.A.J. (2020). Targeting immunometabolism as an anti-inflammatory strategy. Cell Res..

[10] Huo M., Zhang J., Huang W., Wang Y. (2021). Interplay Among Metabolism, Epigenetic Modifications, and Gene Expression in Cancer. Front. Cell Dev. Biol..

[11] Fan J., Krautkramer K.A., Feldman J.L., Denu J.M. (2015). Metabolic regulation of histone post-translational modifications. ACS Chem. Biol..

[12] Frezza C. (2017). Mitochondrial metabolites: Undercover signalling molecules. Interface Focus.

[13] Rubic T., Lametschwandtner G., Jost S., Hinteregger S., Kund J., Carballido-Perrig N., Schwarzler C., Junt T., Voshol H., Meingassner J.G. (2008). Triggering the succinate receptor GPR91 on dendritic cells enhances immunity. Nat. Immunol..

[14] Littlewood-Evans A., Sarret S., Apfel V., Loesle P., Dawson J., Zhang J., Muller A., Tigani B., Kneuer R., Patel S. (2016). GPR91 senses extracellular succinate released from inflammatory macrophages and exacerbates rheumatoid arthritis. J. Exp. Med..

[15] Rubic-Schneider T., Carballido-Perrig N., Regairaz C., Raad L., Jost S., Rauld C., Christen B., Wieczorek G., Kreutzer R., Dawson J. (2017). GPR91 deficiency exacerbates allergic contact dermatitis while reducing arthritic disease in mice. Allergy.

[16] Ridker P.M., Everett B.M., Thuren T., MacFadyen J.G., Chang W.H., Ballantyne C., Fonseca F., Nicolau J., Koenig W., Anker S.D. (2017). Antiinflammatory Therapy with Canakinumab for Atherosclerotic Disease. N. Engl. J. Med..

[17] Tardif J.C., Kouz S., Waters D.D., Bertrand O.F., Diaz R., Maggioni A.P., Pinto F.J., Ibrahim R., Gamra H., Kiwan G.S. (2019). Efficacy and Safety of Low-Dose Colchicine after Myocardial Infarction. N. Engl. J. Med..

[18] Nidorf S.M., Fiolet A.T.L., Mosterd A., Eikelboom J.W., Schut A., Opstal T.S.J., The S.H.K., Xu X.F., Ireland M.A., Lenderink T. (2020). Colchicine in Patients with Chronic Coronary Disease. N. Engl. J. Med..

[19] Tunon J., Badimon L., Bochaton-Piallat M.L., Cariou B., Daemen M.J., Egido J., Evans P.C., Hoefer I.E., Ketelhuth D.F.J., Lutgens E. (2019). Identifying the anti-inflammatory response to lipid lowering therapy: A position paper from the working group on atherosclerosis and vascular biology of the European Society of Cardiology. Cardiovasc. Res..

[20] Berg M., Polyzos K.A., Agardh H., Baumgartner R., Forteza M.J., Kareinen I., Gistera A., Bottcher G., Hurt-Camejo E., Hansson G.K. (2020). 3-Hydroxyanthralinic acid metabolism controls the hepatic SREBP/lipoprotein axis, inhibits inflammasome activation in macrophages, and decreases atherosclerosis in Ldlr-/- mice. Cardiovasc. Res..

[21] Polyzos K.A., Ovchinnikova O., Berg M., Baumgartner R., Agardh H., Pirault J., Gistera A., Assinger A., Laguna-Fernandez A., Back M. (2015). Inhibition of indoleamine 2,3-dioxygenase promotes vascular inflammation and increases atherosclerosis in Apoe-/- mice. Cardiovasc. Res..

[22] Zhang L., Ovchinnikova O., Jonsson A., Lundberg A.M., Berg M., Hansson G.K., Ketelhuth D.F. (2012). The tryptophan metabolite 3-hydroxyanthranilic acid lowers plasma lipids and decreases atherosclerosis in hypercholesterolaemic mice. Eur. Heart J..

[23] Baardman J., Verberk S.G.S., van der Velden S., Gijbels M.J.J., van Roomen C., Sluimer J.C., Broos J.Y., Griffith G.R., Prange K.H.M., van Weeghel M. (2020). Macrophage ATP citrate lyase deficiency stabilizes atherosclerotic plaques. Nat. Commun..

[24] Doddapattar P., Dev R., Ghatge M., Patel R.B., Jain M., Dhanesha N., Lentz S.R., Chauhan A.K. (2022). Myeloid Cell PKM2 Deletion Enhances Efferocytosis and Reduces Atherosclerosis. Circ. Res..

[25] Laurans L., Venteclef N., Haddad Y., Chajadine M., Alzaid F., Metghalchi S., Sovran B., Denis R.G.P., Dairou J., Cardellini M. (2018). Genetic deficiency of indoleamine 2,3-dioxygenase promotes gut microbiota-mediated metabolic health. Nat. Med..

[26] Lu S., Deng J., Liu H., Liu B., Yang J., Miao Y., Li J., Wang N., Jiang C., Xu Q. (2018). PKM2-dependent metabolic reprogramming in CD4(+) T cells is crucial for hyperhomocysteinemia-accelerated atherosclerosis. J. Mol. Med..

[27] Forteza M.J., Berg M., Edsfeldt A., Sun J., Baumgartner R., Kareinen I., Casagrande F.B., Hedin U., Zhang S., Vuckovic I. (2023). Pyruvate dehydrogenase kinase regulates vascular inflammation in atherosclerosis and increases cardiovascular risk. Cardiovasc. Res..

[28] Husted A.S., Trauelsen M., Rudenko O., Hjorth S.A., Schwartz T.W. (2017). GPCR-Mediated Signaling of Metabolites. Cell Metab..

[29] Recio C., Lucy D., Iveson P., Iqbal A.J., Valaris S., Wynne G., Russell A.J., Choudhury R.P., O’Callaghan C., Monaco C. (2018). The Role of Metabolite-Sensing G Protein-Coupled Receptors in Inflammation and Metabolic Disease. Antioxid. Redox Signal..

[30] Li X., Xie L., Qu X., Zhao B., Fu W., Wu B., Wu J. (2020). GPR91, a critical signaling mechanism in modulating pathophysiologic processes in chronic illnesses. FASEB J..

[31] Tang X., Ronnberg E., Safholm J., Thulasingam M., Trauelsen M., Schwartz T.W., Wheelock C.E., Dahlen S.E., Nilsson G., Haeggstrom J.Z. (2022). Activation of succinate receptor 1 boosts human mast cell reactivity and allergic bronchoconstriction. Allergy.

[32] Osuna-Prieto F.J., Martinez-Tellez B., Ortiz-Alvarez L., Di X., Jurado-Fasoli L., Xu H., Ceperuelo-Mallafre V., Nunez-Roa C., Kohler I., Segura-Carretero A. (2021). Elevated plasma succinate levels are linked to higher cardiovascular disease risk factors in young adults. Cardiovasc. Diabetol..

[33] Xu J., Zheng Y., Zhao Y., Zhang Y., Li H., Zhang A., Wang X., Wang W., Hou Y., Wang J. (2022). Succinate/IL-1beta Signaling Axis Promotes the Inflammatory Progression of Endothelial and Exacerbates Atherosclerosis. Front. Immunol..

[34] Consortium G.T. (2013). The Genotype-Tissue Expression (GTEx) project. Nat. Genet..

[35] Pan H., Xue C., Auerbach B.J., Fan J., Bashore A.C., Cui J., Yang D.Y., Trignano S.B., Liu W., Shi J. (2020). Single-Cell Genomics Reveals a Novel Cell State during Smooth Muscle Cell Phenotypic Switching and Potential Therapeutic Targets for Atherosclerosis in Mouse and Human. Circulation.

[36] Alsaigh T., Evans D., Frankel D., Torkamani A. (2020). Decoding the transcriptome of atherosclerotic plaque at single-cell resolution. BioRxiv.

[37] Wirka R.C., Wagh D., Paik D.T., Pjanic M., Nguyen T., Miller C.L., Kundu R., Nagao M., Coller J., Koyano T.K. (2019). Atheroprotective roles of smooth muscle cell phenotypic modulation and the TCF21 disease gene as revealed by single-cell analysis. Nat. Med..

[38] Hao Y., Hao S., Andersen-Nissen E., Mauck W.M., Zheng S., Butler A., Lee M.J., Wilk A.J., Darby C., Zager M. (2021). Integrated analysis of multimodal single-cell data. Cell.

[39] Ma W.F., Hodonsky C.J., Turner A.W., Wong D., Song Y., Mosquera J.V., Ligay A.V., Slenders L., Gancayco C., Pan H. (2022). Enhanced single-cell RNA-seq workflow reveals coronary artery disease cellular cross-talk and candidate drug targets. Atherosclerosis.

[40] Tabula Sapiens C., Jones R.C., Karkanias J., Krasnow M.A., Pisco A.O., Quake S.R., Salzman J., Yosef N., Bulthaup B., Brown P. (2022). The Tabula Sapiens: A multiple-organ, single-cell transcriptomic atlas of humans. Science.

[41] Mulorz J., Spin J.M., Beck H.C., Tha Thi M.L., Wagenhauser M.U., Rasmussen L.M., Lindholt J.S., Tsao P.S.C., Steffensen L.B. (2020). Hyperlipidemia does not affect development of elastase-induced abdominal aortic aneurysm in mice. Atherosclerosis.

[42] Centa M., Ketelhuth D.F.J., Malin S., Gistera A. (2019). Quantification of Atherosclerosis in Mice. J. Vis. Exp..

[43] Yang B., Zhang J., Sun L., Huang T., Kong Y., Li L., Sun Z., Yin M., Li X. (2021). Mapping Novel Biomarkers of Liver Injury by Tissue Proteomic Analysis. ACS Omega.

[44] Bjorklund M.M., Hollensen A.K., Hagensen M.K., Dagnaes-Hansen F., Christoffersen C., Mikkelsen J.G., Bentzon J.F. (2014). Induction of atherosclerosis in mice and hamsters without germline genetic engineering. Circ. Res..

[45] Tomas L., Edsfeldt A., Mollet I.G., Perisic Matic L., Prehn C., Adamski J., Paulsson-Berne G., Hedin U., Nilsson J., Bengtsson E. (2018). Altered metabolism distinguishes high-risk from stable carotid atherosclerotic plaques. Eur. Heart J..

[46] Ketelhuth D.F.J., Lutgens E., Back M., Binder C.J., Van den Bossche J., Daniel C., Dumitriu I.E., Hoefer I., Libby P., O’Neill L. (2019). Immunometabolism and atherosclerosis: Perspectives and clinical significance: A position paper from the Working Group on Atherosclerosis and Vascular Biology of the European Society of Cardiology. Cardiovasc. Res..

[47] De Castro Fonseca M., Aguiar C.J., da Rocha Franco J.A., Gingold R.N., Leite M.F. (2016). GPR91: Expanding the frontiers of Krebs cycle intermediates. Cell Commun. Signal..

[48] Trauelsen M., Hiron T.K., Lin D., Petersen J.E., Breton B., Husted A.S., Hjorth S.A., Inoue A., Frimurer T.M., Bouvier M. (2021). Extracellular succinate hyperpolarizes M2 macrophages through SUCNR1/GPR91-mediated Gq signaling. Cell Rep..

[49] Winther S., Trauelsen M., Schwartz T.W. (2021). Protective succinate-SUCNR1 metabolic stress signaling gone bad. Cell Metab..

[50] Aguiar C.J., Rocha-Franco J.A., Sousa P.A., Santos A.K., Ladeira M., Rocha-Resende C., Ladeira L.O., Resende R.R., Botoni F.A., Barrouin Melo M. (2014). Succinate causes pathological cardiomyocyte hypertrophy through GPR91 activation. Cell Commun. Signal..

[51] Toma I., Kang J.J., Sipos A., Vargas S., Bansal E., Hanner F., Meer E., Peti-Peterdi J. (2008). Succinate receptor GPR91 provides a direct link between high glucose levels and renin release in murine and rabbit kidney. J. Clin. Investig..

[52] Marsal-Beltran A., Rodriguez-Castellano A., Astiarraga B., Calvo E., Rada P., Madeira A., Rodriguez-Pena M.M., Llaurado G., Nunez-Roa C., Gomez-Santos B. (2023). Protective effects of the succinate/SUCNR1 axis on damaged hepatocytes in NAFLD. Metabolism.

[53] Villanueva-Carmona T., Cedo L., Madeira A., Ceperuelo-Mallafre V., Rodriguez-Pena M.M., Nunez-Roa C., Maymo-Masip E., Repolles-de-Dalmau M., Badia J., Keiran N. (2023). SUCNR1 signaling in adipocytes controls energy metabolism by modulating circadian clock and leptin expression. Cell Metab..

[54] Macias-Ceja D.C., Ortiz-Masia D., Salvador P., Gisbert-Ferrandiz L., Hernandez C., Hausmann M., Rogler G., Esplugues J.V., Hinojosa J., Alos R. (2019). Succinate receptor mediates intestinal inflammation and fibrosis. Mucosal Immunol..

[55] Lukasova M., Malaval C., Gille A., Kero J., Offermanns S. (2011). Nicotinic acid inhibits progression of atherosclerosis in mice through its receptor GPR109A expressed by immune cells. J. Clin. Investig..

[56] Pols T.W., Nomura M., Harach T., Lo Sasso G., Oosterveer M.H., Thomas C., Rizzo G., Gioiello A., Adorini L., Pellicciari R. (2011). TGR5 activation inhibits atherosclerosis by reducing macrophage inflammation and lipid loading. Cell Metab..

[57] Miyazaki-Anzai S., Masuda M., Levi M., Keenan A.L., Miyazaki M. (2014). Dual activation of the bile acid nuclear receptor FXR and G-protein-coupled receptor TGR5 protects mice against atherosclerosis. PLoS ONE.

[58] Baumgartner R., Casagrande F.B., Mikkelsen R.B., Berg M., Polyzos K.A., Forteza M.J., Arora A., Schwartz T.W., Hjorth S.A., Ketelhuth D.F.J. (2021). Disruption of GPR35 Signaling in Bone Marrow-Derived Cells Does Not Influence Vascular Inflammation and Atherosclerosis in Hyperlipidemic Mice. Metabolites.

[59] Shewale S.V., Brown A.L., Bi X., Boudyguina E., Sawyer J.K., Alexander-Miller M.A., Parks J.S. (2017). In vivo activation of leukocyte GPR120/FFAR4 by PUFAs has minimal impact on atherosclerosis in LDL receptor knockout mice. J. Lipid Res..

[60] Correa P.R., Kruglov E.A., Thompson M., Leite M.F., Dranoff J.A., Nathanson M.H. (2007). Succinate is a paracrine signal for liver damage. J. Hepatol..

[61] Mills E.L., Pierce K.A., Jedrychowski M.P., Garrity R., Winther S., Vidoni S., Yoneshiro T., Spinelli J.B., Lu G.Z., Kazak L. (2018). Accumulation of succinate controls activation of adipose tissue thermogenesis. Nature.

[62] McCreath K.J., Espada S., Galvez B.G., Benito M., de Molina A., Sepulveda P., Cervera A.M. (2015). Targeted disruption of the SUCNR1 metabolic receptor leads to dichotomous effects on obesity. Diabetes.

[63] Ricci C., Ruscica M., Camera M., Rossetti L., Macchi C., Colciago A., Zanotti I., Lupo M.G., Adorni M.P., Cicero A.F.G. (2018). PCSK9 induces a pro-inflammatory response in macrophages. Sci. Rep..

[64] Jaen R.I., Povo-Retana A., Rosales-Mendoza C., Capillas-Herrero P., Sanchez-Garcia S., Martin-Sanz P., Mojena M., Prieto P., Bosca L. (2022). Functional Crosstalk between PCSK9 Internalization and Pro-Inflammatory Activation in Human Macrophages: Role of Reactive Oxygen Species Release. Int. J. Mol. Sci..

[65] Grebe A., Hoss F., Latz E. (2018). NLRP3 Inflammasome and the IL-1 Pathway in Atherosclerosis. Circ. Res..

[66] Katsuki S., Jha P.K., Lupieri A., Nakano T., Passos L.S.A., Rogers M.A., Becker-Greene D., Le T.D., Decano J.L., Ho Lee L. (2022). Proprotein Convertase Subtilisin/Kexin 9 (PCSK9) Promotes Macrophage Activation via LDL Receptor-Independent Mechanisms. Circ. Res..

