# Supplementary material for: G-Protein-Coupled Receptor 91-Dependent Signalling Does Not Influence Vascular Inflammation and Atherosclerosis in Hyperlipidaemic Mice
Source: Cells. 2023 Nov 6;12(21):2580. doi: 10.3390/cells12212580 (PMC10647868; doi:10.3390/cells12212580)
Supplement: Supplementary file 1 [file cells-12-02580-s001.zip › cells-2663267-SI.pdf]

SUPPLEMENTARY MATERIAL

Supplementary Table S1. List of primers used in the study

Gene target	Forward primer (5' to 3')	Reverse primer (5' to 3')
Cd68	ATC CCC ACC TGT CTC TCT CA	TTG CAT TTC CAC AGC AGA AG
Cd3e	CTG CTA CAC ACC AGC CTC AA	GCC TTC CTA TTC TTG CTC CA
Chil3	CCC TGC CTG TGT ACT CAC CT	GTC CAA ACT TCC ATC CTC CA
Cxcl10	AAG TGC TGC CGT CAT TTT CT	CCT ATG GCC CTA ATT CTC AC
Nos2	ACT GTG TGC CTG GAG GTT CT	TCT CTG CCT ATC CGT CTC GT
Fizz1	CCC TTC TCA TCT GCA TCT CC	AGG AGG CCC ATC TGT TCA TA
Cd206	AGT GAT GGA ACC CCA GTG AC	AGT GGT TGC TCA CAG GCT CT
Arg1	GCA GAG GTC CAG AAG AAT GG	CTG GTT GTC AGG GGA GTG TT
Tnf	CAA AGG GAG AGT GGT CAG GT	ATT GCA CCT CAG GGA AGA GT
Abcc6	TGC GGC CTA TCA CTT GCT C	CCA GCA CCA TTT TGG TTT TGA A
G3bp	TGC TGG TTC CAG GGA CTC AA	CCA CCG GCC TCT GTA GAA GA
Tbp	CAG CCT TCC ACC TTA TGC TC	TTG CTG CTG CTG TCT TTG TT

Supplementary Table S2. Filtering and parameters used for analyses of scRNAseq datasets.

Dataset	Minimum number of genes	Minimum number of genes	Maximum percentage of mitochondrial reads	Principal components	Resolution
GSE131778 (Wirka et al.)	500	3500	7.5	10	0.5
GSE155512 (Pan et al.)	200	4000	10	10	0.15
GSE159677 (Alsaight et al.)	300	4000	10	10	0.5

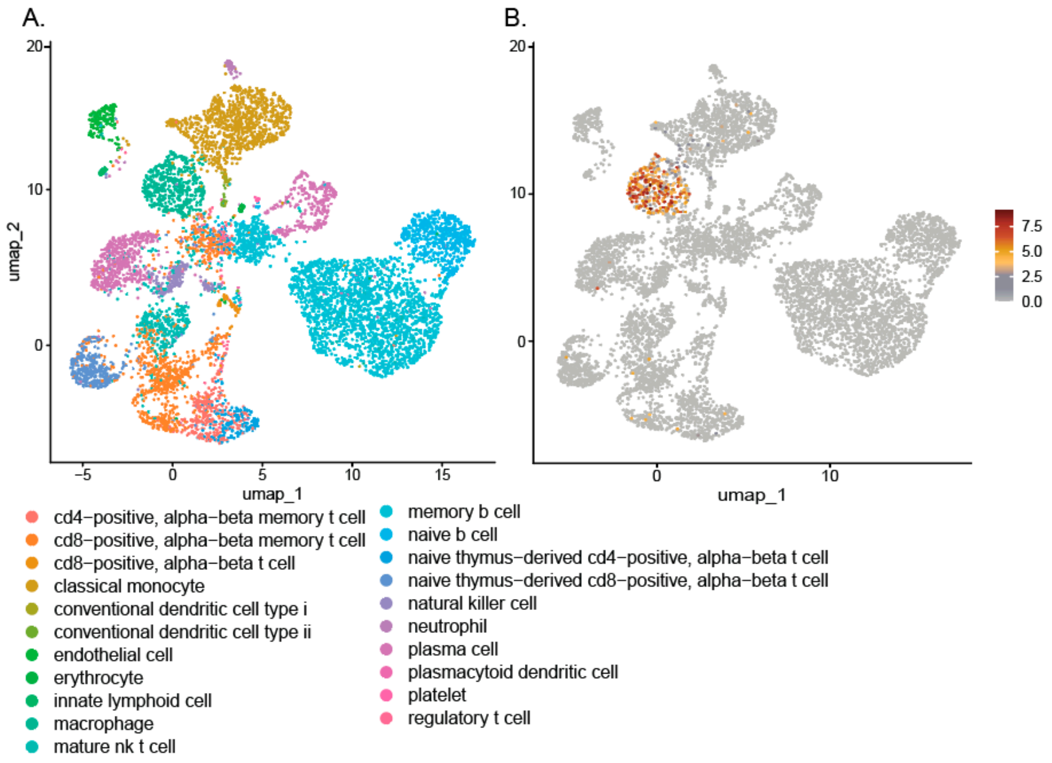

Supplementary Figure S1. *SUCNR1(GPR91)* is expressed mainly in macrophages in the human spleen. A) dimplot showing different cell types in human splenic tissue. B) Feature plot showing expression of *SUCNR1*.

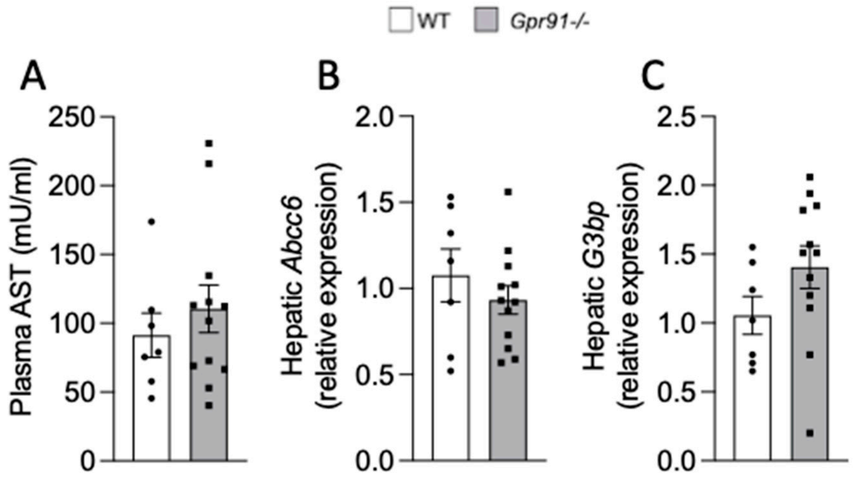

Supplementary Figure S2. Liver toxicity status. Mice were injected with rAAV8-D377Y-mPcsk9 and fed a WTD for 14 weeks. A) AST levels in plasma. B) Relative mRNA expression of associated makers of liver damage *Abcc6* and *G3bp* in the liver from WT and Gpr91-/- mice (n = 7-12). Results are shown as mean ± SEM; graphs show pooled data from two independent experiments. Mann-Whitney U-test shows no differences between groups.
